# Does Character Strength Have an Influence on Children’s Susceptibility to Technological Addiction? A Systematic Review

**DOI:** 10.3390/healthcare14060724

**Published:** 2026-03-12

**Authors:** Ana Jimenez-Perianes, Carlos Monfort-Vinuesa, Elena Saiz-Clar, Maria P. Egea-Romero, Cristina Rebate, Monica Rodriguez-Cañas, Caroline Villarroel, Esther Rincon

**Affiliations:** 1 Psycho-Technology Lab, Universidad San Pablo-CEU, CEU Universities, Urbanización Montepríncipe, 28660 Boadilla del Monte, Spain; a.jimenezp@ceu.es (A.J.-P.); carlos.monfortvinuesa@ceu.es (C.M.-V.); pegea@ceu.es (M.P.E.-R.); cristina.rebate@gmail.com (C.R.); monicamaria.rodriguezcanas@usp.ceu.es (M.R.-C.); caroline.villarroelwesterbarke@usp.ceu.es (C.V.); 2 Departamento de Psicología y Pedagogía, Facultad de Medicina, Universidad San Pablo-CEU, CEU Universities, Urbanización Montepríncipe, 28660 Boadilla del Monte, Spain; 3 Instituto Universitario de Estudios de las Adicciones (IEA-CEU), Universidad San Pablo-CEU, CEU Universities, Urbanización Montepríncipe, 28660 Boadilla del Monte, Spain; 4 Servicio de Medicina Interna, Hospital HM, 28015 Madrid, Spain; 5 Departamento Interfacultativo de Matemática Aplicada y Estadística, Facultad de Ciencias Económicas y Empresariales, Universidad San Pablo-CEU, CEU Universities, Urbanización Montepríncipe, 28660 Boadilla del Monte, Spain; elena.saizclar@ceu.es

**Keywords:** parents, technology, children, character strengths, digital addiction

## Abstract

**Background**: The misuse of technology and the consequences of addiction are topics that are increasingly addressed by researchers. Specifically, more attention is being paid to strategies aimed at preventing such addiction in minors. In all likelihood, this is the first systematic review to focus on the role of moral values in preventing minors from developing digital addictions. The aim of the study is to review the scientific research on the issue in order to answer the following questions: (1) Is character strength (or related variables) linked to an increased probability that minors will develop a technology addiction? (2) What types of character strengths protect minors from these problems? (3) What kinds of training programmes have been provided to instil these values in both minors and parents? **Methods**: The authors systematically examined the peer-reviewed literature from the Web of Science Core Collection (WOS), Medline, and Scopus while adhering to the PRISMA statement. Only articles published until 26 April 2025, and which were written in the English language, were reviewed. The search was conducted with no year restrictions. **Results**: A total of 609 studies were obtained, of which 9 were finally selected. The results provide a complete overview of the presence of moral values as a protective factor against digital addiction. **Conclusions**: Several challenges remain with regard to enhancing the knowledge of moral values, as well as their importance as an instrument available to minors for their safe interaction with technology.

## 1. Introduction

In today’s world, the infamous words of Groucho Marx ring true more than ever before: “Those are my principles! And if you don’t like those, I have others”. The use of new technology by adolescents exposes them to various situations that challenge their principles and moral values. The strength of those values is a hallmark that can protect them from the vastness, volatility, and chameleon-like nature of today’s digital world, and can reinforce their character as well.

Can the moral values of adolescents protect them from misuse of the digital environment? Is it possible for teenagers to avoid the algorithms designed for them by social networks, thanks to their values and principles?

This systematic review aims to analyse recent scientific evidence linking moral values and character strengths to digital safety regarding online use of technology by adolescents and young people. The objective is to identify patterns, gaps in the literature, and opportunities for educational and psychosocial intervention in order to promote healthy moral development in the digital era, which can serve as a protective shield for young people and adolescents.

### 1.1. Digital Addiction in Minors

Digital addiction among minors is a growing concern, with rates at around 3% among adolescents and 11.3% among those in psychiatric hospitals [1]. This behavioural addiction encompasses the misuse of the Internet and computer devices, which requires a multi-faceted approach to diagnosis and treatment [2]. Addiction to social networks can increase anxiety, depression, and sleep disorders in minors [3]. Treatment strategies include preventive measures such as “digital hygiene,” psychotherapy for behavioural addiction and social maladjustment, and psychopharmacotherapy for comorbid conditions such as depression or ADHD [2]. Some consequences of internet addiction, which is characterized by excessive or poorly controlled preoccupations, urges, or behaviors regarding Internet use that lead to impairment or distress in daily functioning [4], include a decline in attentional skills, which in turn reduces their capacity for memory processing [5]; slower language development due to a decrease in parent–child interaction [6]; a loss of creativity and imagination [7]; and other physical impacts such as sleep loss and weight gain, leading to a sedentary lifestyle, which in combination can pose an important risk to children’s health [8]. Protective factors include a cohesive family environment, social skills, an internal locus of control, a strong support system in times of difficulty, satisfying personal relationships, and clear life goals [9]. The misuse of social media can pose other significant cybersecurity risks such as cyberbullying, hate speech, fraud, and privacy violations. The anonymity of the perpetrators and the high rates of victimization make it difficult to resolve these problems [10].

To address these issues, experts recommend promoting the responsible use of social media through open communication, media literacy programs, and content moderation efforts [3]. However, as these approaches might be considered “traditional”, and therefore ineffective, another approach is needed.

### 1.2. The Definition of Character Strengths

In this context, character strengths can be understood as positive and morally valued psychological traits that are reflected in patterns of thought, emotion, and behaviour, and that represent the psychological manifestations of universal human virtues. According to the VIA (Values in Action) [11] classification proposed by the branch of positive psychology, these strengths constitute the observable expressions of six core virtues—wisdom, courage, humanity, justice, temperance, and transcendence—and play a central role in personal development, well-being, and optimal functioning across the lifespan [11].

Moral values play a significant role in the development and dynamics of social networks. Similar behaviours and attitudes among individuals and their peers, especially regarding integrity, are predictors of social distance both online and offline. The analysis of social media content, along with various experiments, indicates that differences in the quality of moral values, more than other aspects of morality, are the best predictors of online bonding [12].

Developing moral values in the family and educational environments results in personal attributes that engender integrity and human growth, or the concept of “strength of character” [13], which is composed of six virtues and 24 character strengths that form the basis of Seligman’s theory of well-being [14].

However, the interaction between morality and social media is complex. While network heterogeneity generally increases openness to attitudinal change, the introduction of morality can often nullify or even reverses this effect [15], which makes critical thinking vital at these moments. The unwillingness to think critically is related to higher levels of perceived stress, which is associated with an increase in problematic smartphone use and less academic interest. Critical thinking has a comprehensive effect on academic engagement, while perceived stress and excessive smartphone use act as intervening factors between critical thinking and academic involvement, thereby fomenting maladaptive coping strategies [16].

Within military units, the features of social networks influence moral reasoning by creating similarities within groups [17]. These findings highlight the intricate relationship between moral values and the social media structure, which emphasizes the importance of taking moral values into account in order to understand social connections and the dynamics of persuasion, both of which operate under the umbrella of critical thinking.

In this regard, moral values play an important role in interaction on social networks and can act as a protective factor against abuse. Various studies have shown that shared moral values, especially those involving integrity, have an influence on social connections and networking [12]. However, social networks embody moral values that might not be transparent to users, which highlights the need to conduct continuous ethical assessments, develop critical thinking skills, question social network content, and discern the quality of interaction on SNs.

To combat online abuse, many social networks have implemented built-in reporting features. The incorporation of these tools by users depends on factors such as perceived responsibility, assumed usefulness, and the fear of judgment. Improvements in cybersecurity on social media are constantly being made. However, the initial protection arises from the individual, which highlights the importance of moral values as part of their toolkit to be used when starting their journey on social networks. Moreover, the role of parents and educators is crucial in preparing young people for their initiation into virtual social interaction.

From an early age, these parental figures and educators transmit moral values as fundamental principles in guiding human behaviour and decision-making in various situations, including within the family, in educational settings, and in the realm of friendship. Five key moral values have been identified by Scott [18], which include the following: honest communication, respect for property, valuing life, regard for religion, and justice. These values can be analysed in terms of aspects such as the act, the actor, the person affected, the intention, and the expected result. In educational settings, moral values are integrated into teaching practices through features such as value transfer, reflective practice, moral sensitivity, dialogue, and critical thinking. Moral values are defined according to the situation in question and can vary among individuals based on factors such as the category, agent, object, effect, and intention of the value in question. Morality implies a complex code of conduct accepted in specific societies and cultures, which reflects prevailing attitudes toward ethical issues, and which guides people toward what they consider to be a good life [12,19].

Moral values play a fundamental role in the social acceptance of technology and in the promotion of safety and social harmony. Values such as privacy, justice, and trust can act as both drivers and barriers to technology acceptance [20]. People’s spiritual and moral development is crucial in overcoming the challenges posed by constant network interaction and the consequences of sharing material that might affect one’s peers [21]. Research on the attitudes of students reveals a combination of consumerist, authoritarian, and constructive perspectives regarding social interaction and safety. Intrinsic factors such as moral maturity and emotional intelligence can positively influence attitudes and behaviour toward safety, which can lead to better outcomes compared to more traditional, extrinsic approaches [22], such as those referred to above regarding technological solutions based on control and surveillance. These findings highlight the importance of considering moral values and personal development when addressing various aspects of young people’s safety, as well as that of their peers, in their social media interaction.

Previous studies on adolescent digital addiction have primarily focused on personality traits, family relationships, behavioral factors, or contextual variables, such as parenting styles, sleep habits, or physical activity, without incorporating moral values or character strengths frameworks.

The “character strength response” has been proposed as a solution to help individuals and society navigate through complex decision-making in an era of technological progress. This concept suggests that character strength, which results from moral values being taught at an early age, leads to an increase in positive emotions, enhanced engagement, better relationships, a meaningful life, and achievement [23]. Consequently, the present study aims to answer the following research questions: (1) Is character strength (or related variables) associated with a higher probability that minors will develop a technological addiction? (2) What kinds of character strengths act as protectors? (3) What types of training programmes have been provided in order to instil these values in both minors and parents?

## 2. Materials and Methods

### 2.1. General Description

A systematic search was carried out on 26 April 2025 to find all relevant studies investigating character strengths (or related variables) as factors that prevent digital addiction in minors. The search was conducted and reported using the statement of Preferred Reporting Items for Systematic Reviews and Meta-Analyses, or PRISMA [24] (see study protocol in Supplementary Information S1, Table S1) [25,26,27,28]. The protocol was also registered with the PROSPERO International Prospective Register of Systematic Reviews (CRD420251041146).

### 2.2. Selection Criteria

Inclusion criteria: The studies were considered relevant if they were journal articles involving character strengths (or related variables) that are measured in either minors or parents with the purpose of promoting healthy technological use by underaged children and adolescents (between 10 to 19 years old). The papers were required to be published in the English language, and they had to provide specific findings, either qualitative or quantitative.

Exclusion criteria: Studies that did not involve character strengths (or related variables), measured in either minors or parents, or were not related to digital use or abuse by minors, were excluded. Research protocols and reviews were also left out, in addition to non-journal articles such as congressional abstracts, book chapters, and theses, due to the primary focus of interest being journal articles, which provided enough specific information in order to properly analyse the primary and secondary objectives of this study.

### 2.3. Outcomes

The main findings of the literature search involved the identification of character strengths (or related variables) and the traits associated with a higher probability that minors will develop a technology addiction. Variables were considered “related variables” when, although not explicitly described as such in the VIA model, they show a high degree of similarity to those included within it. For example, “Family functioning” (defined as the quality of interactional patterns within the family system) can be related to the following VIA strengths: Love, kindness, and teamwork [13]. The search also pinpointed the attributes that act as protective factors, including character traits instilled by parents as a result of parenting styles. The secondary findings were the features related to training programmes developed to promote character strength in minors and parents.

### 2.4. Search Methodology

A comprehensive search was carried out in the Web of Science Core Collection (WOS), Medline, and Scopus on 26 April 2025. The detailed search strategies used for all the databases are provided in Supplementary Information S1. The database search string used was as follows: Abstract (“minors” OR “youth” OR “adolescents” OR “children”) AND (“values” OR “personal values” OR “character strengths” OR “virtues” OR “personal strength” OR “virtue” OR “trait”) AND (“technology” OR “digital” OR “internet” OR “devices”) AND (“abuse” OR “addiction” OR “dependency” OR “misuse”). All original research articles were retrieved for examination, and a search library was created using RefWorks©, a bibliography management program.

### 2.5. Data Collection and Analysis

Two authors (M.R-C. and C.V.) independently evaluated and reviewed all the titles and abstracts for completeness in three phases: Firstly, the titles of the records were assessed; Secondly, the abstracts were evaluated; Thirdly, after reading the titles and abstracts, if a reviewer considered a reference to be relevant, the full text of the paper was extracted. Next, Cohen’s Kappa scores were calculated to measure the inter-rater agreement between the two researchers (M.R-C. and C.V.). The interpretation of Cohen’s Kappa coefficient was calculated using SPSS version 29 (IBM Corp., Armonk, NY, USA) and was based on the categories developed by Douglas Altman [29] as follows: 0.00–0.20 (poor), 0.21–0.40 (fair), 0.41–0.60 (moderate), 0.61–0.80 (good), and 0.81–1.00 (very good). In the case of discrepancies, a third author was consulted (C.R.). Information from the selected studies was retrieved by two authors (C.R. and E.R.). Cross-checking was carried out to identify any inaccuracies or oversights (C.M.-V. and A.J-P.). Any other discrepancies were resolved among the core team with the involvement of the broader research team when necessary.

### 2.6. Data Extraction and Management

We extracted the required data based on (1) publication year, (2) country, (3) study design, (4) study aim, (5) sample size and mean age of the participants, (6) socioeconomic status, (7) character strengths (or related variables), (8) main findings, and (9) training programmes designed to promote character strengths.

### 2.7. Quality of Studies Included

Given the variety of research designs, the quality of the selected studies was assessed using the Mixed Methods Appraisal Tool, or MMAT, which was developed in 2006 [30] and revised in 2018 [31] (see Supplementary Information S2, Table S2). Two authors (E.S-C. and M.P.E.-R.) independently extracted data regarding the results from all the studies. One reviewer (E.R.) examined the data for completeness.

### 2.8. Statistical Analysis

The data were pooled using SPSS version 29 (IBM Corp.), which enabled an analysis of frequencies (percentages), as well as figures regarding the means. Due to the heterogeneity of study designs, outcome measures, and analytical approaches across the included studies, the results are presented using a descriptive and narrative synthesis rather than a quantitative meta-analytic approach.

## 3. Results

### 3.1. Study Selection and Inclusion

**Figure 1:**
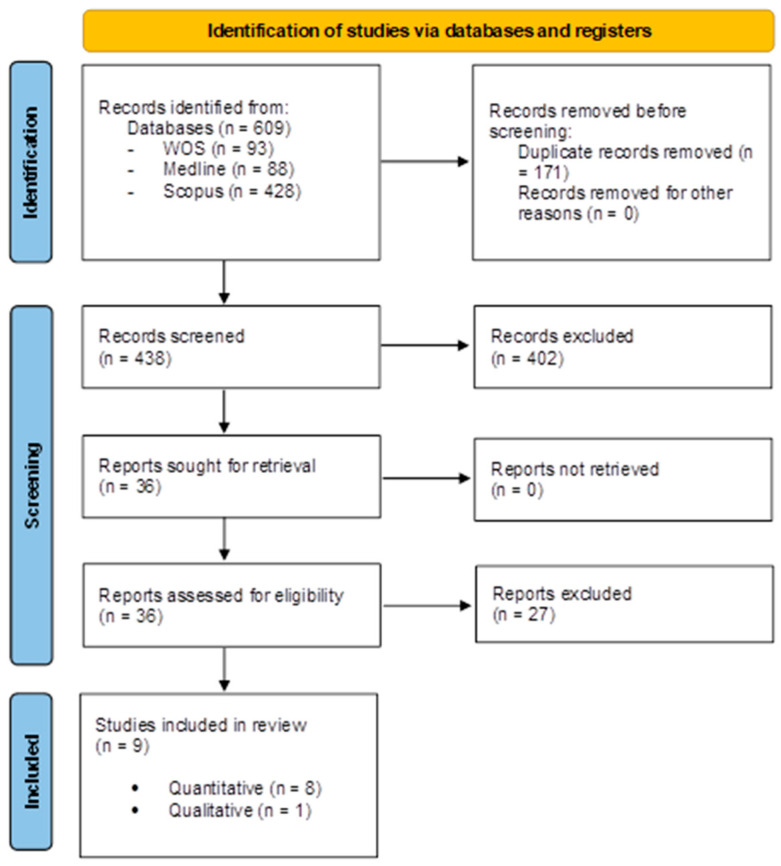
Flowchart of the systematic literature review.

A total of 609 records extracted from the electronic database search were placed in RefWorks©. After removing 171 duplicates, 438 records were evaluated based on the title and abstract. Of those, 402 were removed because they clearly did not meet the inclusion criteria. Thus, 36 papers were selected for a full-text reading, and 27 of those were discarded [32,33,34,35,36,37,38,39,40,41,42,43,44,45,46,47,48,49,50,51,52,53,54,55,56,57,58] for various reasons (see Supplementary Information S3, Table S3). A total of 9 publications [59,60,61,62,63,64,65,66,67] were finally selected. Cohen’s Kappa showed a substantial level of agreement, which was categorized as “good” (κ = 0.77) (range 0.61–0.80), based on the categories developed by Altman [29]. Figure 1 displays a PRISMA flowchart [28]. All the selected studies were deemed to be of sufficient quality in order to contribute equally to the synthesis of the topic.

### 3.2. General Characteristics of the Studies Included

Regarding Point 1 (publication year), P2 (country), and P3 (study design), the following results were obtained (Table 1): The nine selected studies were published between 2016 (n = 1; 11.1%) [63] and 2024 (n = 2) [59,66], with China having the highest number (n = 4; 44.4%) [59,60,62,67] (see Table 1).

Regarding study design, 8 out of 9 papers (88.8%) used a quantitative methodology, often employing cross-sectional data in order to explore correlations between psychological, familial, and behavioural variables, as well as problematic technological use. Only one study [59] took a qualitative and narrative approach, using semi-structured interviews and a thematic analysis in order to explore parenting styles and internet gaming addiction in rural China.

In addressing Point 4 (study aim), P5 (sample size and mean age of the participants), and P6 (socioeconomic status), the results can be seen in Table 2. Although the objectives of the selected studies were similar, they were also varied. The main aim of the chosen studies was to identify psychological, behavioural, and family-related factors associated with problematic or addictive technology use among minors. While all the studies shared this overall objective, they differed in focus and approach. Several investigations concentrated on individual attributes such as empathy and personality traits [61,66] or time management skills [60]. Others prioritized family-related variables, including parenting styles [60,62,64], family dynamics [66], and communication quality [65]. One study used an integrated mediation model, exploring how factors such as physical activity, peer relationships, and self-control mediate the relationship between situational factors and the overuse of technology [67].

Socioeconomic status (SES) was underreported; only two studies provided specific SES information [59,61]. Dalvi-Esfahani et al. [61] compared students from both high- and low-income school settings and found that behaviour associated with Social Media Addiction (SMA) was more prevalent among young people attending high-income schools.

### 3.3. Assessment of Methodological Quality of Included Studies

Even though the majority of the studies used a quantitative design, there was widespread heterogeneity in the statistical methods employed, as well as considerable diversity in the presentation of the results (see Supplementary Information S2, Table S2). The studies included in this review generally utilized large participant samples; however, none specifically focused on individuals with digital addictions, thereby limiting the generalizability of the findings to clinical populations. Methodologically, data collection techniques were predominantly based on psychometric instruments, which enhances the reliability and validity of the measurement of the constructs involved. Furthermore, the data analysis techniques employed were, in general, appropriate for the nature of the data collected.

### 3.4. Primary Outcomes

With regard to Point 7 (character strengths or related variables) and Point 8 (main study findings), the following results were obtained (Table 3). The studies included in this review addressed various psychological, behavioural, and family-related factors associated with problematic or addictive technological use by minors. A prominent topic in multiple studies was the role of parenting styles. Parental emotional care and warmth emerged as a significant preventive factor, showing negative associations with both Problematic Internet Use (PIU; excessive or poorly controlled internet use associated with psychological, social, or functional impairment) [62] and Internet Gaming Disorder (IGD) [60]. Specifically, Chen et al. [60] found that emotional warmth, characterized by displays of parental love and care, reduced IGD symptoms through improved time management, which is a personality trait encompassing autonomy, self-control, and self-reliance in daily behaviour. By contrast, both neglect and overprotection were positively correlated with IGD, yet no significant mediation through time management was found. A partial mediating effect was observed for maternal overprotection, although its overall impact on the model was limited.

In addition, Hayixibayi et al. [62] revealed that higher levels of parental control and lower levels of parental nurturing were significantly associated with an increase in PIU. Moreover, both mother–adolescent and father–adolescent conflict were independently and positively associated with PIU, including various types of disputes such as verbal confrontation, emotional abuse, and even physical punishment. In Bao et al. [59], four parenting styles linked to online gaming addiction were identified: chaotic/conflictive, indulgent, neglectful, and coercive. Each of these styles was associated with children’s emotional detachment, poor behavioural boundaries, and excessive independence, which can lead to compensatory gaming behaviour. The study reported that these parenting styles were not only associated with addiction, but also appeared to be reinforced by it, which is described by the authors as a self-perpetuating cycle.

Other studies broadened this focus by exploring additional aspects of family interaction, such as psychological control, communication quality, and overall cohesion, as key factors in adolescents’ vulnerability to harmful technological use. Lee et al. [63] focused on the effects of psychological control by parents, finding it to be positively associated with mobile phone dependency among adolescents. The authors also discovered that students who perceived high levels of psychological control by their parents also reported less ability to adapt to school settings. The study identified mobile phone dependency as a mediating variable in the relationship between psychological control and school adaptation, while self-regulated learning also played a significant mediating role. According to the authors, intrusive parenting styles not only contribute to excessive mobile phone use, but also impair adolescents’ ability to develop autonomous learning skills, and they can undermine academic engagement as well. Complementing these findings, Monteiro et al. [65] emphasized the role of communication within the family structure.

Beyond family-related factors, several studies focused on individual psychological traits and their role in adolescents’ susceptibility to problematic technological use. Dalvi-Esfahani et al. [61] examined the influence of empathy and personality traits, finding that lower levels of two specific components of empathy, which are Empathic Concern (EC) and Perspective Taking (PT), were associated with higher SMA. The study also analysed the moderating role of personality traits, revealing that extraversion significantly moderated the relationship between both EC and PT, and SMA, thereby reducing the protective effect of empathy as extraversion increased. In Nwufo and Ike [66], internet addiction was found to be positively correlated with extraversion, openness to experience, conscientiousness, and neuroses, yet negatively correlated with the trait of being good-natured. Healthy family interaction, which is seen as the adolescent’s perception of emotional support, adaptability, and cohesion within the family unit, was negatively associated with internet addiction. Moreover, family interaction played a moderating role, as it strengthened the preventive effect of good-naturedness and weakened the negative impact of extraversion on internet addiction.

Finally, one of the studies highlighted the importance of physical activity in developing individual preventive factors against internet addiction. Qiu et al. [67] showed that physical activity had an indirect negative effect on internet addiction through its positive influence on self-control, self-reliance, and psychological resilience. These variables acted as mediators in a multiple intermediary model, suggesting that strengthening internal regulatory and motivational abilities might reduce adolescents’ vulnerability to excessive internet use.

**Figure 2:**
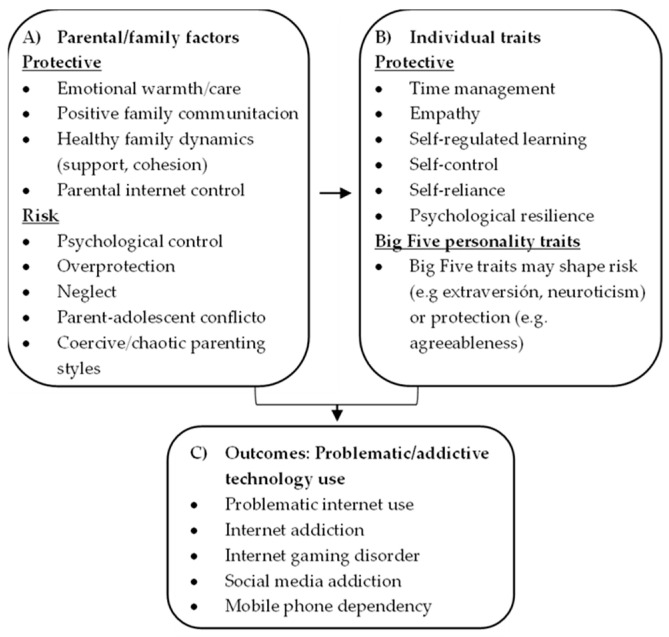
Conceptual map summarizing the main relationships identified across the included studies.

Figure 2 presents a conceptual map summarizing the main relationships identified between parenting and family factors, individual traits, and problematic or addictive technology use in minors.

### 3.5. Secondary Outcomes

With regard to Point 9 (training programmes designed to promote character strength), none of the studies included in this review implemented or tested specific prevention or intervention strategies targeting harmful technological use (Table 4).

## 4. Discussion

Previous studies have addressed the importance of developing moral values in children and adolescents [12,18], in addition to the connection between these virtues and the new technologies [12,13,14,15,16,17,18] in preventing the development of digital addiction. However, to the best of our knowledge, this is the first systematic review aimed at examining character strengths and values developed within the family unit and their association with addiction to new technologies, as well as the main benefits—understood as preventive factors in digital addiction interventions—and the challenges related to the use of these strengths in the management of digital tools. This evidence can be associated with the character strengths model proposed by Peterson and Seligman, which organizes 24 strengths into six virtues and posits that they function as protective psychological resources, particularly relevant in high-risk contexts such as digital addictions [13].

The variables analyzed in the included studies were explicitly aligned with specific VIA character strengths [11,13], based on conceptual and functional overlap rather than strict terminological equivalence. Variables such as emotional absence, rigid control, and lack of psychological support—comparable to dysfunctional parenting styles—were associated with deficits in the VIA strengths of love, kindness, and social intelligence (Humanity), as well as fairness (Justice). In contrast, emotional warmth was aligned with the strength of love, while overprotection and parental control were conceptually linked to prudence and self-regulation (Temperance), and effective time management was associated with self-regulation and perspective (Wisdom). Finally, self-control, self-reliance, and psychological resilience were conceptually associated with self-regulation, perseverance, hope, and bravery, highlighting the relevance of VIA character strengths as protective resources within family contexts related to digital risk and technology use [13].

The review focused on analysing recent scientific evidence that links moral values, character strengths, and digital safety with regard to children’s use of social media.

The findings of this systematic review highlight the significant influence of the family environment, as well as certain psychological traits of individuals, on adolescents’ vulnerability to troubling or addictive technological use. The results can be interpreted by applying the character strength model proposed by Peterson and Seligman [13], which focuses on developing qualities and traits for building healthy relationships [68].

### 4.1. Individual Traits

One of the strengths identified in several studies is self-regulation, which is part of the virtue of temperance. Both time management and self-control emerged as key factors that mediate between parenting styles and the risk of Internet Gaming Disorder (IGD) or internet addiction [60,67]. Within the character strengths framework, self-regulation influences impulse inhibition and frustration tolerance in response to highly persuasive digital stimuli, which explains its strong protective role.

This suggests that fostering self-regulation in adolescents, along with affection and autonomy, may act as protective factors against harmful technological use.

Likewise, as self-reliance is related to the strength of perseverance, this trait was also identified as an important mediating factor. According to Bandura [69], self-reliance influences the way people confront challenges, regulate their behaviour, and develop resilience when their endeavours are unsuccessful. Within the Peterson and Seligman framework, self-reliance can be linked to both Courage (perseverance) and Transcendence, as it facilitates minors’ ability to cope with difficulties without resorting to compulsive technology use as a means of escape [13].

This ability to experience a feeling of competence in the face of daily challenges reduces the need to resort to technology as a form of escape or compensation, which also underscores the child’s resilience. The empowerment of these strengths is nurtured in family environments that foster trust, positive communication, and responsible independence.

On the other hand, there are other strengths as well. One is love, which is expressed through emotional support and parental accompaniment, and empathy, which is related to the virtue of love and humanity in the model illustrated by Peterson and Seligman [13]. Moreover, both of these traits are associated with lower levels of addiction to social media and the Internet [61]. Eisenberg et al. [70] pointed out that in addition to fostering healthy interpersonal relationships, empathy is associated with a lower frequency of disruptive behaviour and higher emotional self-regulation, which validates its role as a preventive factor against addictive behaviour. These observations are consistent with the virtue of Humanity within the character strengths model, which explains how the strengthening of affective bonds and interpersonal sensitivity translates into reduced reliance on technology as an external regulator of emotions.

### 4.2. Parenting Skills

Based on the parenting skills that shape different virtues in children, a significant relationship has been found between the strength of wisdom and knowledge (expressed by the love of learning and critical thinking) and the use of new technology. As pointed out by Rincon et al. [71], the teaching of parenting skills is essential for preventing and reducing the addiction to new technology by minors. Likewise, according to Lee et al. [63], psychological control by parents increases problematic mobile phone use. In addition, children with strong self-regulation skills develop better cognitive, motivational, and behavioural control strategies, which are crucial for avoiding distractions such as the compulsive use of digital devices, as suggested in the study by Zimmerman [72]. Within the Peterson and Seligman framework, strengths such as judgment and critical thinking enable minors to interpret digital information more appropriately, identify potential risks, and guide their decisions in a constructive manner, thereby acting as protective factors against the influence of persuasive digital algorithms [13].

### 4.3. Protective Factors

Regarding a strong sense of justice, when families maintain rules and boundaries based on fairness, this creates a family environment that encourages the proper use of technology [65,66]. The perception of justice is a predictor of emotional balance in adolescence [73], which reinforces its protective value in high-risk situations such as those to be found in the domain of new technology.

Physical activity also appears to be a protective factor against addiction, as it promotes temperance, or self-regulation, as well as the strength of perseverance and vitality. This association is supported by Lubans et al. [74], who showed that physical activity not only improves a person’s bodily health but their mental health as well by increasing psychological well-being, self-esteem, and resilience.

### 4.4. Strength Based Prevention or Intervention Activities

Finally, although none of the studies recommended specific prevention or intervention programmes based on developing the character strengths and values set forth by Peterson and Seligman [13], Jimenez-Perianes [68] has proposed various hands-on activities that can be developed at home based on the same model. Likewise, in a study conducted by Lavy [75], school programmes were implemented that focused on strengthening values, improving students’ well-being, and reducing problematic behaviour in minors. Furthermore, similar initiatives that focus on families and minors with regard to preventing or treating addiction to new technology should be encouraged. This need is even greater if we consider the published findings that verify deficits in emotional regulation and inhibitory control in adolescents with video game disorder, as well as the lack of psychosocial interventions aimed at addressing the stigma associated with this behaviour in the family environment [76].

### 4.5. Training Strategies and Benefits

Autonomy based on critical thinking rather than impulsive behaviour can act as a protective factor against internet addiction, and it is important to design intervention programmes aimed at developing these discernment skills in minors. The concept of justice is paramount in interaction with digital networks, and it is necessary to differentiate justice from the binary model “fair/unjust” through learning programs aimed at situations appropriate to the age of the minors. Physical activity is also a preventive factor. The promotion of sporting activities, either individual or collective, should be included as part of the programmes mentioned above.

### 4.6. Challenges to Address

One of the main challenges for the future is to reach agreement on the need for moral values and character strength so that parents can find the best ways to communicate these virtues to their children. Of course, parental figures must also understand that they too are at risk of falling victim to a technology addiction, which could prevent them from transmitting the values essential for healthy technological use to their children.

#### Brief Proposal for an Ideal Intervention

In light of the findings, an effective intervention should be grounded in three fundamental pillars: self-regulation, empathy and emotional regulation, and family communication and structure, articulated through the virtues proposed by the Peterson and Seligman model [13]. First, sessions aimed at minors should include practical strategies such as the establishment of graduated goals, the design of healthy digital routines, and the identification of early-warning signs of loss of control, thereby strengthening the virtue of Temperance. Second, the program should incorporate activities focused on identifying and understanding emotions, analyzing social situations, practicing empathic responses, and developing assertive communication skills, complemented by critical digital literacy content linked to the virtue of Wisdom. Finally, working with families is essential to improve communication processes, promote parenting styles based on warmth and autonomy, and establish clear and equitable rules regarding technology use. This component reinforces the virtue of Justice by fostering a predictable and coherent environment that supports minors’ self-regulation. In addition, as a cross-cutting complement, the promotion of structured physical activity is recommended, given its protective role in enhancing self-regulation, perseverance, and resilience.

## 5. Conclusions

Based on the proposed objectives and the results obtained from the systematic review, the following conclusions have been reached:

1. The empirical evidence confirms that character strengths such as emotional warmth, autonomy, self-control, critical thinking, empathy, perseverance, vitality, and resilience, which are associated with positive parenting styles, act as protective factors against problematic technological use in children. By contrast, psychological control by parents and a lack of values can increase the likelihood of developing digital addictions. These effects appear to be primarily mediated by self-regulation (the virtue of Temperance) and by processes of emotional regulation and perspective-taking (the virtue of Humanity), which highlights clear targets within the character strengths model for the development of prevention frameworks in family and school contexts.
2. Given the diverse approaches, Seligman’s character strengths, which include temperance, humanity, and wisdom, offer a useful framework for understanding and addressing the problem of technological addiction from the viewpoint of the family and the positive development of minors. This provides different targets for multicomponent interventions focused on behavioral models, socioemotional skills, digital literacy, and the establishment of clear norms.
3. As the systematic review did not find any specific prevention or intervention protocols that integrate these protective factors in families, this is a priority that clearly demands further research.
4. Based on the results, a strengths map can be outlined—comprising self-regulation, empathy, critical thinking, perseverance, perceived justice, and vitality—that interacts with parenting practices (warmth, autonomy support, and positive communication) to reduce the likelihood of compulsive use; this pattern supports a preventive approach grounded in the Peterson and Seligman model [13].
5. The absence of validated strengths-based protocols in family and school contexts reveals a priority opportunity for the development of multicomponent interventions and their rigorous evaluation.

### 5.1. Clinical and Researcher Implications

Randomized controlled trials are needed in the future in order to address the questions that remain, which are the following: Would the development of protocols aimed at fostering moral values or character strengths be effective in reducing the incidence of digital addiction among minors? Might the effectiveness of such protocols depend on the degree of prior abuse or addiction in the minor, or perhaps on certain personality traits or age-related variables? Would it be effective to teach parents how to incorporate these virtues into their parenting strategies? Should teachers also be included in these protocols so that they can promote these values in the classroom?

In any case, further studies are needed, not only to answer these questions, but also to improve the knowledge regarding the effectiveness of such programs.

From a clinical perspective, these findings suggest that professionals should systematically assess strengths such as self-regulation, empathy, perseverance, and the quality of family communication, as well as recommend physical activity, given that these factors function as protective elements against problematic technology use.

Psychological interventions could incorporate intervention targets associated with this model to develop preventive programs or therapeutic approaches for minors at risk of digital addiction.

### 5.2. Limitations

Regarding the limitations of this study, the first is the small number of studies that associate moral values with addiction to new technology among minors. Moreover, the very definition of moral values can be confused with character strength if the language of origin is taken into account.

Likewise, with regard to cultural variability in moral values, the high proportion of studies conducted in Chinese populations may introduce a cultural bias that limits the external validity of the findings. This geographical concentration reduces the ability to generalize the results to other cultural contexts with potentially different value systems.

In addition, an imbalance between qualitative and quantitative studies was observed, with only one qualitative study included alongside eight quantitative studies. Although the inclusion criteria allowed for qualitative and mixed-methods research in order to capture clinically relevant information aligned with the initial research objectives, the final body of evidence was predominantly quantitative. This imbalance may limit the extent to which qualitative perspectives are represented in the synthesis of findings and should be taken into account when interpreting the results.

## Figures and Tables

**Table 1:** General characteristics of included studies (n = 9).

Study	Publication Year	Country	Study Design
Bao et al. [59]	2024	China	Qualitative
Chen et al. [60]	2020	China	Quantitative
Dalvi-Esfahani et al. [61]	2021	Iran	Quantitative
Hayixibayi et al. [62]	2022	China	Quantitative
Lee et al. [63]	2016	Republic of Korea	Quantitative
Martins et al. [64]	2020	Portugal	Quantitative
Monteiro et al. [65]	2023	Portugal	Quantitative
Nwufo and Ike [66]	2024	Nigeria	Quantitative
Qiu et al. [67]	2023	China	Quantitative

**Table 2:** General Features of the Selected Studies (II) (n = 9).

Study	Purpose of the Study	Sample Size (Mean Age)	Socioeconomic Status
Bao et al. [59]	Examine parenting styles and online game addiction in rural children.	41 adolescents (NP. Aged between 12–14); 20 teachers and 14 parents	Rural and low-income families lacking cultural capital.
Chen et al. [60]	Examine how parenting styles and time management can predict internet gaming disorders in adolescents.	357 adolescents (NP)	NP
Dalvi-Esfahani et al. [61]	Examine the influence of empathy and personality traits on social media addiction among high school students.	592 adolescents (16.46)	Students from high-income and low-income schools.
Hayixibayi et al. [62]	Evaluate parent–adolescent conflict and parenting styles as risk factors in problematic internet use by adolescents.	6552 adolescents (NP. Aged between 10–19 years)	NP
Lee et al. [63]	Assess the impact of psychological control by parents on children’s school life, with mobile phone dependency as a mediator.	2378 children (10.2)	NP
Martins et al. [64]	Determine the prevalence of internet addiction among adolescents and assess the role of parental control.	1916 adolescents (15 ± 1.8 years)	NP
Monteiro et al. [65]	Explore the links between internet addiction, sleep habits, and family communication in adolescents.	340 adolescents (14.55)	NP
Nwufo and Ike [66]	Examine the moderating role of family dynamics in the relationship between personality traits and internet addiction among adolescents.	3150 adolescents (15.7)	NP
Qiu et al. [67]	Test a multiple, intermediary model of adolescent physical exercise and internet addiction.	466 adolescents (NP. Aged between 14–18)	NP

Abbreviations: NP = Not provided by the authors.

**Table 3:** Primary outcomes (n = 9).

Study	Character Strengths (or Related Variables)	Main Findings
Bao et al. [59]	Emotional absence, rigid control, and a lack of psychological support (outlined above as comparable to the four parenting styles).	Four distinct parenting styles in which the failure to teach strength-related skills was linked to online gaming addiction.
Chen et al. [60]	Emotional warmth, overprotection, and neglect (parenting styles), and time management.	Emotional warmth was negatively correlated with gaming disorders through better time management. Both neglect and overprotection were associated with an increase in internet gaming disorders, but time management showed no mediating effect.
Dalvi-Esfahani et al. [61]	Empathy, personality traits (the Big Five).	Low-level empathy was associated with higher rates of social media addiction. Extraversion moderates the relationship.
Hayixibayi et al. [62]	Parental care (warmth, empathy), parental control (overprotection, intrusion), and parent–adolescent conflict.	Parent–adolescent conflict and parental control were positively associated with problematic internet use, yet higher parental care had a negative association.
Lee et al. [63]	Psychological control by parents, self-regulated learning.	Psychological control by parents was associated with higher mobile phone dependency, poor adaptation to the school setting, and a low level of self-regulated learning.
Martins et al. [64]	Parental internet control.	Parental internet control was inversely related to internet addiction; well-being was negatively affected by internet addiction.
Monteiro et al. [65]	Family communication.	Poor family communication and daytime sleepiness were linked to increased internet addiction.
Nwufo and Ike [66]	Personality traits (the Big Five), family dynamics (problem-solving ability, communication, and emotional closeness).	Internet addiction was positively associated with neuroses, extraversion, conscientiousness and openness; and negatively associated with agreeableness and positive family dynamics. Family interaction played a moderating role (agreeableness and extraversion).
Qiu et al. [67]		More physical activity predicts higher self-control, mental resilience, and self-reliance, contributing to lower levels of internet addiction.

**Table 4:** Secondary outcomes (n = 9).

Study	Training Developed
Bao et al. [59]	NP
Chen et al. [60]	NP
Dalvi-Esfahani et al. [61]	NP
Hayixibayi et al. [62]	NP
Lee et al. [63]	NP
Martins et al. [64]	NP
Monteiro et al. [65]	NP
Nwufo and Ike [66]	NP
Qiu et al. [67]	NP

Abbreviations: NP, Not provided by the authors.

## Data Availability

No new data were created or analyzed in this study. Data sharing is not applicable to this article.

## Supplementary Materials

The following supporting information can be downloaded at: https://www.mdpi.com/article/10.3390/healthcare14060724/s1, Supplementary Information S1—study protocol; Supplementary Information S2—quality assessment of the selected studies (MMAT); Supplementary Information S3—reasons for the exclusion of certain studies.
