# Supplementary material for: Does Character Strength Have an Influence on Children’s Susceptibility to Technological Addiction? A Systematic Review
Source: Healthcare (Basel). 2026 Mar 12;14(6):724. doi: 10.3390/healthcare14060724 (PMC13027014; doi:10.3390/healthcare14060724)
Supplement: Supplementary file 1 [file healthcare-14-00724-s001.zip › supporting information_S1_ER_03.03.26.pdf]

Supplementary Information, S1

Adopted from PRISMA-P (Preferred Reporting Items for Systematic review and Meta-Analysis Protocols) and PROSPERO.

Table S1. Study protocol.

**Topic**	**Content**
Title	Does Character Strength have an Influence on Minors' Susceptibility to Technological Addiction? A Systematic Review.
Authors	Ana Jimenez-Perianes, Carlos Monfort-Vinuesa, Elena Saiz-Clar, Maria P. Egea-Romero, Cristina Rebate, Monica Rodriguez-Cañas, Caroline Villarroel and Esther Rincon
Review team members and their organizational affiliations	• **Prof. Ana Jimenez-Perianes,** PhD.<br>1. Psycho-Technology Lab (Universidad San Pablo-CEU, CEU Universities).<br>2. Departamento de Psicología y Pedagogía, Facultad de Medicina, Universidad San Pablo-CEU, CEU Universities.<br>3. Instituto Universitario de Estudios de las Adicciones (IEA-CEU), Universidad San Pablo-CEU, CEU Universities.<br>• **Prof. Carlos Monfort-Vinuesa,** PhD.<br>1. Psycho-Technology Lab (Universidad San Pablo-CEU, CEU Universities).<br>2. Departamento de Psicología y Pedagogía, Facultad de Medicina, Universidad San Pablo-CEU, CEU Universities.<br>4. Servicio de Medicina Interna. Hospital HM. Madrid<br>• **Prof. Elena Saiz-Clar,** PhD.<br>5. Facultad de Ciencias Económicas y Empresariales, Departamento Interfacultativo de Matemática Aplicada y Estadística, Universidad San Pablo-CEU,

	CEU Universities, Urbanización Montepríncipe, 28660 Boadilla del Monte, España<br><br>• **Prof. Maria P. Egea-Romero**, PhD.<br>1. Psycho-Technology Lab (Universidad San Pablo-CEU, CEU Universities).<br>2. Departamento de Psicología y Pedagogía, Facultad de Medicina, Universidad San Pablo-CEU, CEU Universities.<br>• **Ms Cristina Rebate** MA.<br>1. Psycho-Technology Lab (Universidad San Pablo-CEU, CEU Universities).<br>• **Ms Monica Rodriguez-Cañas**.<br>1. Psycho-Technology Lab (Universidad San Pablo-CEU, CEU Universities).<br>• **Ms Caroline Villarroel**<br>1. Psycho-Technology Lab (Universidad San Pablo-CEU, CEU Universities).<br>• **Prof. Esther Rincon,** PhD.<br>1. Psycho-Technology Lab (Universidad San Pablo-CEU, CEU Universities).<br>2. Departamento de Psicología y Pedagogía, Facultad de Medicina, Universidad San Pablo-CEU, CEU Universities.<br>3. Instituto Universitario de Estudios de las Adicciones (IEA-CEU), Universidad San Pablo-CEU, CEU Universities.
Contact details of corresponding author	Prof. Esther Rincón<br>Psycho-Technology Lab (Universidad San Pablo-CEU).<br>Departamento de Psicología y Pedagogía, Facultad de Medicina, Universidad San Pablo-CEU, CEU Universities,<br>Urbanización Montepríncipe,<br>28660 Boadilla del Monte, España.

	Phone: +34 913 724 700.<br>Email: maria.rinconfernande@ceu.es
Organizational affiliation of the review	Psycho-Technology Lab<br>(Universidad San Pablo-CEU, CEU Universities),<br>Madrid (Spain).
Type and method of review	Systematic review
Contributions	Ana Jimenez-Perianes (A.J-P.): Conceptualization, Formal analysis, Investigation, Project administration, Resources, Supervision, Validation, Visualization, Writing – original draft, Writing – review and editing. Carlos Monfort-Vinuesa (C M.): Formal analysis, Investigation, Project administration, Supervision, Validation, Visualization, Writing – original draft, Writing – review and editing. Elena Saiz-Clar (E.S-C): Conceptualization, Data curation, Formal analysis, Investigation, Methodology, Software, Writing – original. Maria P. Egea-Romero (M.P. E-R): Conceptualization, Data curation, Formal analysis, Investigation, Methodology, Software, Writing – original draft, Writing – review and editing. Cristina Rebate (C.R.): Conceptualization, Data curation, Formal analysis, Software, Visualization, Writing – original draft, Writing – review and editing. Monica Rodriguez-Cañas (M.R.-C.): Data curation, Formal analysis, Software, Visualization, Writing – original draft. Caroline Villarroel (C.V.): Data curation, Formal analysis, Software, Visualization, Writing – original draft. Esther Rincon (ER): Conceptualization, Formal analysis, Funding acquisition, Investigation, Methodology, Project administration, Resources,

	Supervision, Validation, Visualization, Writing – original draft, Writing – review and editing. All the authors gave the final approval of the version to be published, and they agreed to be accountable for all aspects of the work by ensuring that questions related to the accuracy or integrity of any part of the study were appropriately investigated and resolved. All the authors have read and agreed to the published version of the manuscript.
Sources/Sponsors	This research received funding from Fundación Universitaria San Pablo-CEU (CEU Universities).
Conflict of interest	Authors declare no conflict of interest.
Rationale	The objectives of this study were as follows;<br>1) to determine if character strength (or related variables) are linked to an increased probability that minors develop a technology addiction.<br>2) to detect the kind of character strengths that protect minors from these problems.<br>3) to establish the main training programs developed to instill these values in both minors and parents.
Eligibility criteria	Inclusion criteria:<br>(1) Primary research studies (journal articles) involving character strengths (or related variables), which are measured in either minors or parents, the purpose of which is to promote healthy technological use by their underaged children.<br>(2) Published in English and providing specific findings (qualitative or quantitative results).<br>Exclusion criteria:<br>(1) Studies that did not involve character strengths (or related variables), measured in either minors or parents, or were not related to digital use or abuse by minors.

	(2) Protocols with unpublished results, narratives reviews, no journal articles (conference Proceeding, book chapters or Thesis).<br>(3) Other language than English.
Information sources	A systematic review was conducted, based on scientific literature on April 26th, 2025 in Web of Science Core Collection (WOS), Medline, and Scopus.
Search strategy	Search strategy for all the database search:<br>Filters: English language; no year restrictions.<br>Conducted on April 26th, 2025.<br>The results were extracted with the keywords:<br>Abstract: ("minors" OR "youth" OR "adolescents" OR "children") AND ("values" OR "personal values" OR "character strengths" OR "virtues" OR "personal strength" OR "virtue" OR "trait") AND ("technology" OR "digital" OR "internet" OR "devices") AND ("abuse" OR "addiction" OR "dependency" OR "misuse").
Type of included study	Only Journal Articles.
Studied domain	Behavioral Addictions
Population/Participants	Parents and minors (under 18 years old)
Data collection and selection process	The original versions of all the research articles were retrieved for examination, and a search library was created using RefWorks©. All authors substantially contributed to analysis, data interpretation, and revised the work critically. All extracted data were reviewed for completeness by A.J.-P and ER. All authors gave final approval of the version to be published and agreed to be accountable for all aspects of the work by ensuring that questions related to the accuracy or integrity of any part of the work are appropriately investigated and resolved.
Data items for coding	The following data were extracted from the selected articles: (1) publication year, (2) country, (3) study design, (4) study aim, (5) sample size and mean age of the

	participants, (6) socioeconomic status, (7) character strengths (or related variables), (8) main findings, and (9) training programs designed to promote character strengths.
Outcomes and prioritization	The primary outcomes involved the identification of character strengths (or related variables), and the traits associated with a higher probability that minors will develop a technology addiction. The search also pinpointed the attributes that act as protective factors, including character traits instilled by parents as a result of parenting styles. The secondary findings were the features related to training programs developed to promote character strength in minors and parents.
Data synthesis	The PRISMA statement will be followed for data synthesis, and a narrative synthesis of the included studies will be performed.
Language	English.
Country	Spain.
Anticipated or actual start date	April 26th, 2025
Anticipated or actual end date	July 2025.

Adopted from Booth et al [25], Moher et al [26], Page et al [27] and Shamseer et al [28].
