# Supplementary material for: Does Character Strength Have an Influence on Children’s Susceptibility to Technological Addiction? A Systematic Review
Source: Healthcare (Basel). 2026 Mar 12;14(6):724. doi: 10.3390/healthcare14060724 (PMC13027014; doi:10.3390/healthcare14060724)
Supplement: Supplementary file 1 [file healthcare-14-00724-s001.zip › supporting information_S2_ER_03.03.26.pdf]

Table S2. Quality of studies included: Mixed Methods Appraisal Tool (MMAT) [30-31].

Type of study	Study	Screening questions		Quantitative randomized controlled trials				
		S1. Are there clear research questions?	S2. Do the collected data allow to address the research questions?	1.1.Is the qualitative approach appropriate to answer the research question?	1.2. Are the qualitative data collection methods adequate to address the research question?	1.3. Are the findings adequately derived from the data?	1.4. Is the interpretation of results sufficiently substantiated by data?	1.5. Is there coherence between qualitative data sources, collection, analysis and interpretation?
59. Bao et al. (2024)	Qualitative	Yes	Yes	Yes	Yes	No	No	Yes
60. Chen et al. (2020)	Quantitative	Yes	Yes					
61. Dalvi-Esfahani et al. (2021)	Quantitative	Yes	Yes					
62. Hayixibayi et al. (2022)	Quantitative	Yes	No					
63. Lee et al. (2016)	Quantitative	Yes	Yes					
64. Martins et al. (2020)	Quantitative	Yes	Yes					
65. Monteiro et al. (2023)	Quantitative	Yes	Yes					
66. Nwufo and Ike (2024)	Quantitative	Yes	Yes					
67. Qiu et al. (2023)	Quantitative	Yes	Yes					

Table S2. Quality of studies included: Mixed Methods Appraisal Tool (MMAT) [30-31].

		Quantitative descriptive studies				
Type of study	Study	2.1. Is the sampling strategy relevant to address the research question?	2.2. Is the sample representative of the target population?	2.3. Are the measurements appropriate?	2.4. Is the risk of nonresponse bias low?	2.5. Is the statistical analysis appropriate to answer the research question?
59. Bao et al. (2024)	Qualitative					
60. Chen et al. (2020)	Quantitative	Yes	Yes	Yes	Yes	Yes
61. Dalvi-Esfahani et al. (2021)	Quantitative	Yes	Yes	Yes	Yes	Yes
62. Hayixibayi et al. (2022)	Quantitative	Yes	Yes	Yes	Yes	Yes
63. Lee et al. (2016)	Quantitative	Yes	Yes	Yes	No	Yes
64. Martins et al. (2020)	Quantitative	Yes	Yes	Yes	Yes	Yes
65. Monteiro et al. (2023)	Quantitative	Yes	Yes	Yes	Yes	Yes
66. Nwufo and Ike (2024)	Quantitative	Yes	Yes	Yes	Yes	Yes
67. Qiu et al. (2023)	Quantitative	Yes	Yes	Yes	Yes	Yes
