# Supplementary material for: Does Character Strength Have an Influence on Children’s Susceptibility to Technological Addiction? A Systematic Review
Source: Healthcare (Basel). 2026 Mar 12;14(6):724. doi: 10.3390/healthcare14060724 (PMC13027014; doi:10.3390/healthcare14060724)
Supplement: Supplementary file 1 [file healthcare-14-00724-s001.zip › supporting information_S3_ER_03.03.26.pdf]

Supporting Information S3.

Table 3. Reasons for studies exclusion.

Excluded	Reason for exclusion
32. Aral & Usta (2022)	Focuses on adolescents with ADHD, not general adolescent population.
33. Ayten & Acat (2019)	Examines internet addiction as a variable influencing life satisfaction, not as the primary outcome.
34. Bilge et al. (2022)	Does not include variables related to character strengths in parents or minors.
35. Cacioppo et al. (2019)	Does not include variables related to character strengths in parents or minors.
36. Casaló & Escario (2019)	Does not include variables related to character strengths in parents or minors.
37. Chen et al. (2015)	Does not include variables related to character strengths in parents or minors.
38. Ding et al. (2014)	Does not include variables related to character strengths in parents or minors.
39. Fomichov & Fomichova (2012)	Does not provide specific qualitative or quantitative results.
40. Ge et al. (2015)	Does not include variables related to character strengths in parents or minors.
41. Juthamanee & Gunawan (2021)	Does not include variables related to character strengths in parents or minors.
42. Kaya et al. (2024)	Explores how digital games influence creativity, rather than examining creativity as a protective factor influencing digital use.
43. Kuss et al. (2013)	Does not include variables related to character strengths in parents or minors.
44. Lee et al. (2019)	Investigates problematic internet use and life stressors as risk factors for psychotic-like experiences in adolescents
45. Lee et al. (2014)	Focuses on comorbidities and behavioral symptoms associated with internet use.
46. Li et al. (2023)	Does not include variables related to character strengths in parents or minors.
47. Malaeb et al. (2020)	Does not include variables related to character strengths in parents or minors.
48. Mastrobattista et al. (2024)	Does not include variables related to character strengths in parents or minors.
49. Mishra et al. (2024)	Does not include variables related to character strengths in parents or minors.
50. Müller (2017)	Analyzes media influence on minors' values and education; does not assess digital use or character strengths.
51. Munno et al. (2016)	Does not include variables related to character strengths in parents or minors.
52. Peris et al. (2020)	Does not include variables related to character strengths in parents or minors.
53. Riany & Saadatunnisa (2022)	Analyzes how internet addiction negatively affects respect; does not assess respect as a protective character strength.
54. Satria et al. (2024)	Does not include variables related to character strengths in parents or minors.

55. Sun & Wilkinson (2020)	Does not include variables related to character strengths in parents or minors.
56. Trumello et al. (2018)	Does not meet age criteria.
57. Xiuqin et al. (2010)	Does not include variables related to character strengths in parents or minors.
58. Yan et al. (2014)	Does not meet age criteria.
